# The Association Between Muscle Strength, Thigh Circumference, and Serving Efficiency in Elite Male Volleyball Players: A Prospective Observational Longitudinal Study

**DOI:** 10.3390/jfmk11030362

**Published:** 2026-09-09

**Authors:** Leila Itani, Elisa Berri, Oscar Berti, Arianna Ferrentino, Dana Saadeddine, Marwan El Ghoch

**Affiliations:** 1 Department of Nutrition and Dietetics, Faculty of Health Sciences, Beirut Arab University, Riad El Solh, Beirut P.O. Box 11-5020, Lebanon; l.itani@bau.edu.lb; 2 Degree Course of Dietetics, Innovation and Research Training Service, Azienda Ospedaliero-Universitaria di Modena, 41124 Modena, Italy; elisa.berri@unimore.it (E.B.); 321814@studenti.unimore.it (A.F.); 3 Azienda Unità Sanitaria Locale-IRCCS di Reggio Emilia, Department of Primary Care, 42123 Reggio Emilia, Italy; 4 Department of Performance, Modena Volley Club, 41122 Modena, Italy; oscarberti@virgilio.it; 5 Center for the Study of Metabolism, Body Composition and Lifestyle, Department of Biomedical, Metabolic and Neural Sciences, University of Modena and Reggio Emilia, 41125 Modena, Italy; dana.saadeddine@unimore.it

**Keywords:** volleyball, elite athletes, mediterranean diet, muscle strength, thigh circumference, bioelectrical impedance vector analysis (BIVA), serving efficiency, linear mixed models, athletic performance, body composition, diet quality, handgrip strength, anthropometry, team sports

## Abstract

**Background and Aim**: The identification of factors that optimize competitive performance is a primary focus for athletes and coaches in any sport, particularly variables that are modifiable through tailored nutritional and training strategies. Since data regarding volleyball remain scarce, this investigation aimed to explore the association between the baseline preseason anthropometry, body composition, and dietary habits of elite adult male volleyball players and the key performance indicators (KPIs) retrieved as match-by-match data during an official tournament. **Methods**: Nineteen elite male volleyball players (mean age: 23.79 ± 5.72 years; BMI: 23.88 ± 1.97 kg/m^2^) underwent comprehensive assessments. Anthropometric data and muscle strength (MS) were evaluated via body circumferences and handgrip dynamometry, respectively. Body composition was analyzed using bioelectrical impedance vector analysis (BIVA), while Mediterranean diet adherence was assessed with the validated Mediterranean diet adherence score (MEDAS) questionnaire, all measured cross-sectionally at one time point, identified as the baseline preseason phase of the competitive season. The tournament KPIs included serving, attack, and reception efficiencies, and block points per set were retrieved longitudinally during each match of the competitive season. Data were analyzed utilizing correlation and linear mixed-model regressions. **Results**: Right-hand MS correlated positively with higher MEDAS scores (ρ = 0.488, *p* < 0.05). Furthermore, both right-hand MS (β = 1.005, 95% CI [0.178, 1.831], *p* = 0.026) and thigh circumference (β = 3.030, 95% CI [0.083, 5.977], *p* = 0.046) were found to be positively associated with higher serving efficiency (adjusted R^2^_m_ ≈ 0.12). **Conclusions**: Monitoring muscle strength and thigh circumference alongside targeted nutritional and training strategies may be considered in elite male volleyball, as these factors were associated with serving efficiency. However, due to the observational nature of the research, our findings require replication in larger, multicenter intervention studies.

## 1. Introduction

In professional sports, training serves as the foundational period for optimizing an athlete’s physical and mental preparedness to achieve peak competitive performance [1]. Consequently, identifying modifiable factors during training that directly translate into enhanced athletic outcomes is a primary objective for athletes, coaches, and staff across all sport disciplines [2].

In elite volleyball, key performance indicators (KPIs) represent essential collective metrics utilized by coaching staff to evaluate team tactical efficiency and predict match outcomes [3]. Modern scouting systems typically categorize these KPIs into two macro-systems: the side-out (reception system) and the breakpoint (serving and defensive system) phases [4]. Among these, the most critical metrics in professional volleyball include attack (AE), reception (RE), and serving (SE) efficiencies, as well as block points per set (BS) [5].

Specifically, AE exhibits the highest direct correlation with victory, accurately predicting the winning team in over 85% of matches by measuring net offensive production while penalizing unforced errors and blocked hits [6]. SE evaluates the net impact of the collective team serve and acts as a direct predictor of match outcomes; a higher SE restricts the opponent’s offensive options, shifts the tactical advantage to the defending team, and significantly increases winning probability [7]. Similarly, BS is a decisive defensive metric—calculated as total terminal blocks divided by total sets played—representing net actions that yield direct points [8]. Finally, advanced data models utilize RE as a primary metric to predict set winners and final point differentials [9]. Based on these considerations and given the tactical importance of these metrics (KPIs), identifying easily measurable and monitorable factors associated with subsequent performance before the competitive season is of paramount relevance [10].

With this aim, scientific research in elite and competitive volleyball started to shift toward implementing comprehensive preseason baseline testing within an integrated, multidimensional framework. This was initially driven by the published literature that revealed the important role of early preseason baseline assessments in subsequent injury prevention and mitigation during official competitions [11,12,13]. Moreover, some recent studies have suggested that preseason early indicators may also influence, correlate with, or actively predict the main above-mentioned KPIs [14]. Of these, some anthropometric (i.e., height) and body composition (i.e., fat-free mass) variables seem to predict attack performance in high-level male volleyball players [15]. In addition, certain nutritional strategies appear to improve physical performance and sport-specific skills in competitive volleyball players [16]. However, there remains a notable paucity of empirical data and longitudinal tracking directly linking multifaceted preseason baselines with actual in-season game statistics and matchplay efficiency [17].

Therefore, this study aims to investigate the prospective associations between a wide range of preseason anthropometric parameters as well as body composition, nutritional status, and competitive KPIs in elite male volleyball players.

## 2. Materials and Methods

### 2.1. Study Design and Participants

A comprehensive testing protocol encompassing anthropometric measurements, body composition analyses, dietary assessments, and physical performance evaluations was implemented. The sample consisted of 19 elite male volleyball players from ‘Modena Volley’ (Modena, Italy) who volunteered to participate. The Modena Volley team, based in Modena, is the most successful professional men’s volleyball club in Italy and has played in the top Italian division without interruption since 1968, winning 12 national league titles and 12 national cups. Eligible participants were required to be healthy professional athletes with no medical contraindications for elite-level volleyball training and competition; no further exclusion criteria were applied. No formal a priori sample size power analysis was performed because this prospective study included all eligible players from a single elite volleyball team during one competitive season.

Enrolment and baseline testing took place during preseason phase immediately before the initiation of the competitive season. The cohort comprised 14 athletes from the SuperLega roster (the top-tier professional division of the Italian Volleyball League) and five from the Serie B team (the primary national development roster, predominantly composed of elite athletes aged under 19).

The study protocol was reviewed and approved on 19 November 2025 by the Institutional Review Board (Ethics Committee for Research) of the University of Modena and Reggio Emilia (Protocol No. 284220). Prior to initiation, all participants received detailed information regarding the experimental procedures and provided written informed consent. A maximum of 31 weekly matches was played over a duration of seven months between November 2025 and June 2026. The number of matches and sets contributed by each player varied according to match participation. The data for each player in terms of number of matches and sets played are presented in Table S1.

Two trained dietitians from the Center for the Study of Metabolism, Body Composition and Lifestyle, of the Department of Biomedical, Metabolic and Neural Sciences of the University of Modena and Reggio Emilia, were directly involved in the study and conducted all of the following cross-sectional assessments only once at the baseline time point, identified as the preseason phase of the competitive season.

### 2.2. Socio-Demographic Variables

Age, education level, marital status, occupation, nationality, and smoking habits, etc., were retrieved through individual interviews.

### 2.3. Body Weight and Height

The body weight of each participant was measured to the nearest 0.1 kg, using a mechanical weighing scale (SECA 861-7021099, seca GmbH & Co. KG, Hamburg, Germany), and height was assessed to the nearest 0.5 cm with a stadiometer (SECA 225-1721009, seca GmbH& Co. KG, Hamburg, Germany) [18]. Body mass index (BMI) was then determined according to the standard formula of body weight (kg) divided by the square of the height (m^2^) [19].

### 2.4. Body Circumferences and Muscle Strength Performance

In the body circumference assessment, the mid-upper-arm circumference (MUAC), waist circumference (WC), thigh circumference (TC), and calf circumference (CC) were measured with a tape to the nearest 0.1 cm [18].

Muscle strength (MS) was assessed by means of handgrip strength, according to international guidelines [20]. The handgrip strength test was performed on both hands using a calibrated Jamar^®^ Hydraulic Hand Dynamometer (5030J1, Chicago, IL, USA), and the maximum value (kg) was recorded [21].

### 2.5. Body Composition

The bioelectrical impedance analysis was conducted with a phase-sensitive impedance device (BIA 101 BIVA PRO, Akern Systems, Firenze, Italy) in compliance with the manufacturer’s guidelines. Before the measurement, each participant rested in a supine position for one minute and emptied their bladder [22]. All measurements were carried out in a temperature-controlled room. The resistance (Rz), reactance (Xc), and impedance (Z) were taken as raw measurements that were used by the BODYGRAM^®^ HBO ver 2.0 (Akern Systems, Firenze, Italy) to calculate/estimate the following variables:

- Total fat mass (FM): the total fat mass of the total body weight expressed in kg.
- Total fat mass percentage (FM%): the total fat mass in kg of the total body weight in kg expressed in a percentage.
- Total fat-free mass (FFM): the total fat-free mass of the total body weight expressed in kg.
- Total fat-free mass percentage (FFM%): the total fat-free mass in kg of the total body weight in kg expressed in a percentage.
- Total body water (TBW): the total amount of body water expressed in liters.
- Phase angle (PhA°): arctangent (Xc/R) × (180°/π) expressed in degrees (°).

### 2.6. Diet Quality

The Mediterranean diet adherence score (MEDAS) was retrieved from a validated 14-item questionnaire widely used to measure how closely an individual follows and adheres to the Mediterranean diet [23]. Each of the 14 items is scored ‘1’ if criteria are met and ‘0’ if not. The item scores are summed up to give a score ranging from 0 to a maximum possible score of 14. A higher score indicates greater adherence to the Mediterranean diet and hence better overall diet quality. Adherence categories are classified into low (0–5), medium (6–9), and high adherence (≥10). For the current study, categorization was limited by the sample size, hence, the continuous scale was utilized to retain between-player variability. The questionnaire was originally developed for the landmark Prevención con Dieta Mediterránea (PREDIMED) clinical trial in Spain; it evaluates both food quantities and specific culinary habits.

### 2.7. Key Performance Indicators (KPIs)

The KPIs were retrieved longitudinally by the strength and conditioning coach from the Department of Performance of the Modena Volley team directly involved in the study, during each match of the competitive season, using professional software (Data Project, Data Volley, Version 4, computer software, Genus Sports 2020. https://www.dataproject.com/Products/US/en/Volleyball/DataVolley#, accessed on 4 September 2026):

- Serving efficiency (SE%) is an advanced KPI [24] that measures a net positive impact behind the service line by balancing point scoring against unforced errors expressed as a percentage:

SE% = [(Aces + Positive Serves) − Errors]/Total Serves × 100

- Reception efficiency (RE%) is a KPI [25] that measures the ability to handle the opponent’s serve and successfully transition into an offensive attack expressed as a percentage:

RE% = [(Excellent + Positive Passes) − Reception Errors]/Total Reception Attempts × 100

- Attack efficiency (AE%) is an important KPI [6] that independently predicts the wins of the match, expressed as a percentage:

[Kills − (Errors + Blocked)]/Total Attempts × 100

- The blocks per set metric (BS) is a KPI [8] that expresses the number of blocks in a set and is considered a highly reliable KPI of defensive stability and opponent frustration:

Total Winning Blocks/Total Played Sets.

Values for SE%, RE%, and AE% were taken directly from “Data Volley” and not recalculated by the authors. Negative efficiency values occur when the total errors exceed positive actions in each match; consequently, the mean efficiency values may also be negative.

### 2.8. Statistical Analysis

Descriptive statistics are presented as means and standard deviations for continuous variables and frequencies and proportions for categorical variables. The normality of continuous variables was checked using the Shapiro–Wilk test. Given the relatively small sample size and the non-normal distribution of several variables, Spearman’s rank correlation coefficients (ρ) were initially adopted as an exploratory analysis to examine the relationship between the main KPIs and preseason player-level measures including body circumferences and composition parameters, as well as MS and adherence to the Mediterranean diet. They were also employed to identify candidate variables for subsequent linear mixed-effects modeling. Due to the hierarchal structure of performance data with repeated match-level observations nested within individual players, the mixed-effects linear model analyses were subsequently utilized. The number of match-level observations varied across players because not every player participated in all matches; linear mixed models (LMMs) accommodate this unbalanced repeated-measure structure. The use of linear mixed models (LMMs) accounts for the non-independence of the repeated observations obtained from the same player while simultaneously modeling both within- and between-player variability.

Random intercepts were specified for each player (Player ID) to account for the correlation among repeated observations within individuals. Player ID was specified as the primary random effect, whereas additional random effects for match or opponents were not included given the limited sample size and imbalanced participation of players across matches. Associations identified in the exploratory Spearman analysis were considered for subsequent LMM evaluation based on statistical evidence, biological plausibility, and relevance to volleyball performance. Next, a priori adjustment variables (player role and training years) were selected to account for positional differences and playing experience. Confounding was evaluated by comparing regression coefficients (β) between the unadjusted and adjusted models. Covariates were retained in the final model regardless of their statistical significance, given that their removal would cause more than 15–20% change in the regression coefficient of the body composition parameter of interest [26]. Model assumptions of linear mixed-effects models were assessed by examining residual normality, linearity, homoscedasticity, multicollinearity, and influential observations, as well as the distribution of random effects using graphical diagnostic procedures. As a sensitivity analysis, the robust linear mixed-effects function in R was also utilized to determine the influence of atypical observations on the estimated associations [27].

The KPIs for each match were obtained from professional software (Data Project, Data Volley, Version 4, computer software, Genus Sports 2020. https://www.dataproject.com/Products/US/en/Volleyball/DataVolley#, accessed on 4 September 2026). For descriptive statistics and correlation analysis, player-level mean SE, RE, and AE were calculated by summing all efficiencies from the official match-specific efficiency values attained from the software for each match and dividing by the total number of matches played. The number of blocks per set was computed by summing the total blocks for each player from each match and dividing by the total number of sets played. For correlation analyses and all linear mixed-effects models, match-level KPI rows corresponding to non-participation were excluded.

Statistical significance was set at *p* < 0.05. The statistical analysis was performed using IBM SPSS software version 27 (IBM Corp., Armonk, NY, USA, 2020) and RStudio version 2026.07.1 (Posit Software, PBC, Boston, MA, USA).

## 3. Results

The study included 19 elite male volleyball players with a mean age of 23.79 ± 5.72 years and an average of nine years of training (8.58 ± 5.78 years). Each player participated on average in 15.5 ± 7.9 (1 to 27) matches and 48.3 ± 33.3 (2 to 84) sets during the competitive season (Table S1). Their player role mostly varied between spiker (36.8%) or middle blocker (26.3%), followed by libero (15.8%), opposite (10.5%), or setter (10.5%). They were mostly professional volleyball players (73.7%) of Italian nationality (63.2%), not married (84.2%), and non-smokers (63.2%), with a mean MEDAS of 7.21 ± 1.51 (Table 1).

The anthropometric characteristics of the volleyball players are shown in Table 2. The players had a mean height of 196.6 ± 7.9 cm, a mean weight of 92.4 ± 10.5 kg, and a mean BMI of 23.9 ± 1.9 kg/m^2^. The players exhibited a MUAC, TC, CC, and WC of 33.0 ± 2.1 cm, 59.33 ± 3.75 cm, 38.66 ± 3.08 cm, and 83.4 ± 4.7 cm, respectively. The mean MS was 55.8 ± 11.9 kg in the right hand and 55.21 ± 9.56 kg in the left (Table 2).

The Spearman’s rank correlation coefficients are presented in Table 3. No KPIs correlated with anthropometric or body composition parameters except for SE%, which showed a significant association with TC (0.514, *p* < 0.05), CC (0.573, *p* < 0.05), and RH-MS (0.555, *p* < 0.05). MEDAS was significantly associated with RH-MS (0.488, *p* < 0.05) but not LH-MS (0.206, *p* > 0.05). MEDAS was negatively linked with BS (−0.517, *p* < 0.05).

The results of the linear mixed model (LMM) are presented in Table 4. The SE% analysis included 16 players, as three liberos did not contribute to valid serve efficiency according to their player role. These players contributed a total of 227 repeated observations across 31 scheduled matches in the competitive season. The median number of observations per player was 18.5 (IQR 6.25–21; range: 1–27). Player role and training years were included as a priori adjustment variables to account for positional differences and playing experience. Adjustment for these variables substantially increased the estimates (RH-MS and LH-MS: 0.566 to 1.005; TC: 1.640 to 3.030). The strengthened estimate after adjustment supports an independent positive relationship between RH-MS, TC, and SE%. Diagnostic evaluation indicated no major violation of model assumptions. Estimates from the LMM for the relationship between MEDAS and BS were not statistically significant.

Graphical diagnostics procedures for the linear mixed-effects model assumption for both models, RH-MS and TC, indicated no major violations. To assess the robustness of the reported association with these assumptions, a sensitivity analysis was conducted using a robust linear mixed-effects model. Estimates for all 16 player-level random effects from these models were comparable in magnitude and direction and significance to the primary model estimates for RH-MS (β: 1.008 vs. 1.005; *p* < 0.05) and TC (β: 2.853 vs. 3.030; *p* < 0.05), supporting the robustness of the reported association. Furthermore, all 16 player-level random effects received robustness weights approximately equal to one, indicating that no individual player exerted undue influence on the model estimates.

## 4. Discussion

The current study aimed to provide benchmark data on the potential relationships between anthropometric parameters, dietary variables, and body composition as well as KPIs in elite male volleyball players. The analysis revealed three main findings.

### 4.1. Findings and Concordance with Previous Studies

Our first finding indicates that greater adherence to the Mediterranean diet, reflected by higher MEDAS scores, correlates with increased RH-MS. However, due to the cross-sectional assessment—measured at the same time—of these two variables, no cause–effect relationship can be established between MEDAS and RH-MS [28]. Therefore, the hypothesis that superior diet quality is associated with higher MS requires robust confirmation through future longitudinal interventional studies [29]. Nevertheless, our result closely aligns with a recent systematic review by Bianchi et al. [30] on athletes from various sport disciplines, which highlighted that higher adherence to the Mediterranean diet correlates with higher handgrip MS. Critically, while Bianchi et al.’s pool included intervention trials spanning diverse athletic disciplines and non-professional cohorts, the potential relationship between diet quality and muscle strength via dietary antioxidants and anti-inflammatory pathways holds strong biological plausibility for elite male athletes, who face exceptionally high training volumes and severe exercise-induced oxidative stress during the preseason [30]. However, our findings reflect a cross-sectional association within a small cohort of 19 elite athlete.

Our second finding demonstrates that a higher MS assessed in the very early season stage is significantly associated with a better SE% during the subsequent official competition. This highly consistent finding is in line with the previously published literature on MS measured by the handgrip test, which found that it is strongly associated with improved athletic performance in athletes in general [20]. This is likewise the case in volleyball, where despite the paucity of research, handgrip MS has been identified as a vital indicator of overall upper-body power and ball velocity. For instance, while earlier data from Koley and Kaur [31] and Camacho-Villa [32] identified handgrip MS as a vital indicator of upper-body power, it is critical to note that their cohorts consisted exclusively of junior and senior female volleyball players. Therefore, our finding in this regard requires further confirmation before drawing firm conclusions.

Similarly, the third outcome of our investigation reveals a positive association between larger TC and higher SE during the championship. In the volleyball literature, there has traditionally been a paucity of data regarding the direct association between regional anthropometric variables such as TC on in-game technical metrics. The few available studies, such as the one conducted by Jiange et al., utilized ultrasonography on elite male volleyball players and confirmed that the cross-sectional area of the vastus lateralis muscle in the lower limb—of which TC is a surrogate measure—is primarily a predictor of explosive lower-body power and vertical jump height in volleyball [33]. However, the modest marginal R^2^ of the adjusted models indicates that these preseason characteristics explain only a limited proportion of the variability in match-level SE, highlighting the multifactorial nature of volleyball performance.

### 4.2. Potential Implications

Our findings may have important implications for the sporting context, particularly in disciplines such as volleyball. Given that MS and TC are significantly positively associated with SE%, and considering that both parameters are easily, affordably, and safely monitorable over time, routine tracking of these variables alongside nutritional strategies, such as—the Mediterranean diet—could be considered during athlete physical profiling. This information and these findings should be shared and thoroughly discussed with athletes, coaches, and staff. However, due to the observational nature of the research, this potential practical association should be considered a correlational pattern that requires confirmation in larger, multicenter intervention studies.

### 4.3. Study Strengths and Limitations

Our study has several strengths. First, to the best of our knowledge, it is one of the very few to investigate the associations between KPIs and various anthropometric parameters, dietary variables, and body composition in elite male volleyball players. Second, body composition was assessed via BIVA, which exhibits high precision [34], and MS was evaluated using the Jamar^®^ Hydraulic Hand Dynamometer, currently considered a widely accepted instrument for measuring maximal isometric handgrip strength in sports performance settings [35]. Third, the longitudinal design allowed for the examination of associations between MS, TC, and athletic performance (KPIs, specifically SE%), after adjusting for potential confounders [36]. Finally, the use of a ‘mixed-model analysis’—that accounts for the non-independence of repeated observations within the same player while simultaneously modeling both intra- and inter-player variability—should be considered a strong point [37].

On the other hand, our investigation also has certain limitations. First and foremost, the sample size was small; however, this is common in research involving elite athletes [38], as recruiting a large sample in highly specialized sports disciplines is extremely challenging [39]. Second, participants were recruited from a single volleyball club, which may introduce selection bias and further limit the external validity of the findings. In addition, no formal a priori power analysis was conducted. The relatively narrow distribution of the MEDAS score may have limited our ability to detect associations with performance indicators. Third, the inclusion of only male athletes from a specific discipline limits the external validity of our findings, as they cannot be generalized to female players in the same sport or to athletes in other sporting disciplines. Fourth, although adjustment was performed for player role and years of training, residual confounding cannot be excluded. Finally, given the exploratory nature of the correlation analysis, there is an increased risk of false positive findings; therefore, these associations should be interpreted cautiously and require confirmation in larger studies.

### 4.4. New Directions for Future Research

Before drawing definitive practical conclusions, future research is warranted to address the following gaps. First, although our findings revealed an association between higher MEDAS scores and MS, this does not imply a relationship, and we cannot conclude that an increase in Mediterranean diet adherence necessarily leads to improvements in MS.

Second, even if a link exists, future investigations should test whether the improvements in MS that enhance athletic performance—specifically SE%—are mediated by the Mediterranean diet, or if other factors are at play. In this direction, future interventional studies are required to identify which strategies can best optimize MS and TC within training programs.

Third, although we did measure a wide range of variables, we cannot rule out the existence of other factors such as biochemical tests or others that we did not assess but that may affect the KPIs. Finally, because the findings of this study were limited to our specific population of elite Italian male volleyball players, further research is warranted to validate these results in female athletes, as well as in males across different age groups and ethnicities.

## 5. Conclusions

Our findings highlight significant associations between key competition performance indicators (i.e., SE%) and parameters such as RH-MS and TC, which can be easily, safely, and repeatedly monitored. This suggests a hypothesis that structured training programs may play a role in optimizing these variables, opening promising avenues for future interventional studies evaluating athletic performance. However, before considering these findings as practical recommendations, confirmations are required through large sample, multicenter interventional studies. Consequently, such results can assist coaches in tracking specific, personalized physical profiles to monitor MS and body dimensions (i.e., TC), thereby potentially informing athlete development.

## Figures and Tables

**Table 1:** Sociodemographic, lifestyle, dietary, and positional characteristics of male volleyball players (n = 19) *.

Characteristic	Values *
Age (years)	23.8 (5.7)
Marital status	
Single	16 (84.2%)
Married	3 (15.8%)
Nationality	
Italian	12(63.2%)
Other	7(36.8%)
Occupation	
Professional volleyball player	14 (73.7%)
Student and volleyball player	5 (26.3%)
Smoking habits	
Smoker	6 (31.6%)
Non-smoker	12 (63.2%)
Occasionally/sometimes	1 (5.3%)
MEDAS	7.2 (1.5)
Role of each player	
Setter	2 (10.5%)
Spiker	7 (36.8%)
Middle blocker	5 (26.3%)
Opposite	2 (10.5%)
Libero	3 (15.8%)

* Values are means (SD) for continuous variable and n (%) for categorical variables.

**Table 2:** Anthropometric, body composition, physical performance characteristics and volleyball performance indicators of the male volleyball players (n = 19).

Characteristic	Minimum	Maximum	Mean (SD)
Height (cm)	181.0	206.6	196.6 (7.9)
Weight (kg)	74.7	111.3	92.4 (10.5)
BMI (kg/m^2^)	19.7	27.4	23.9 (1.9)
MUAC (cm)	28.5	36.0	33.0 (2.1)
TC (cm)	52.4	65	59.3 (3.8)
CC (cm)	31.6	44	38.7 (3.1)
WC (cm)	74.5	93.8	83.4 (4.7)
PhA (°)	5.7	8	7.1 (0.6)
FFM (kg)	65.6	90.0	78.4 (7.5)
FFM (%)	80.9	89.1	85.1 (2.3)
FM (kg)	9.1	21.3	13.9 (3.5)
FM (%)	10.9	19.1	14.9 (2.3)
TBW (L)	46.3	61.5	54.6 (4.8)
RH-MS (kg)	42.0	90	55.8 (11.9)
LH-MS (kg)	39	70	55.2 (9.6)
SE (%)	−66.7	39.5	−3.5 (22.2)
RE (%)	−50	100	38.5 (28.2)
AE (%)	−50	66.2	24.3 (30.8)
BS	0	0.6	0.3 (0.2)

BMI = body mass index; MUAC = middle-upper arm circumference; TC = thigh circumference; CC = calf circumference; WC = waist circumference; PhA° = phase angle; FFM = fat free mass; FFM% = fat free mass percentage; FM = fat mass; FM% = fat mass percentage; TBW = total body water; RH-MS = right hand muscle strength; LH-MS = left hand muscle strength; SE% = serving efficiency; RE% = reception efficiency; AE% = attack efficiency; BS = blocks per set.

**Table 3:** Spearman correlation coefficients between the volleyball performance indicators and anthropometric, body composition, physical performance and dietary parameters among male volleyball players (n = 19).

Body Composition/Lifestyle	SE (%)	RE (%)	AE (%)	BS
MUAC (cm)	0.440	0.274	0.145	−0.241
TC (cm)	0.514 *	0.300	0.352	−0.125
CC (cm)	0.573 *	0.229	0.303	0.103
WC (cm)	0.313	0.229	0.094	0.183
FFM (%)	−0.476	0.152	−0.265	−0.166
FM (%)	0.476	−0.152	0.265	0.166
TBW (L)	0.169	0.434	0.235	0.109
RH-MS (kg)	0.555 *	−0.035	−0.056	0.027
LH-MS (kg)	0.470	−0.171	−0.086	0.281
MEDAS	0.115	0.171	0.03	−0.517 *

MUAC = middle-upper arm circumference; TC = thigh circumference; CC = calf circumference; WC = waist circumference; FFM% = free fat mass percentage; FM% = fat mass percentage; TBW = total body water; RH-MS = right hand muscle strength; LH-MS = left hand muscle strength; SE% = serving efficiency; RE% = reception efficiency; AE% = attack efficiency; BS = blocks per set; MEDAS = Mediterranean Diet Adherence Score. * Correlation was significant at *p* < 0.05 level (2-tailed).

**Table 4:** Unadjusted and adjusted estimates of linear mixed models for the relation between body composition, physical performance and dietary parameters, and volleyball performance indicators *.

		Unadjusted LMM					Adjusted LMM *				
Parameter	Outcome	β	95% CI	*p* Value	R^2^_m_	R^2^_c_	β	95% CI	*p* Value	R^2^_m_	R^2^_c_
RH-MS (kg)	SE%	0.566	−0.178; 1.311	0.106	0.056	0.182	1.005	0.178; 1.831	0.026	0.118	0.225
TC (cm)	SE%	1.640	−0.596; 4.236	0.145	0.046	0.155	3.030	0.083; 5.977	0.046	0.129	0.266
CC (cm)	SE%	2.065	−3.332; 7.460	0.179	0.055	0.108	2.920	−0.670; 6.512	0.090	0.117	0.280
MEDAS score	BS	−0.059	−0.138; 0.021	0.134	0.032	0.171	−0.049	−0.134; 0.036	0.229	0.107	0.174

LMM = linear mixed model; TC = thigh circumference; CC = calf circumference; RH-MS = right hand muscle strength; * Models adjusted for player role and training years; MEDAS = Mediterranean Diet Adherence Score; * R^2^_m_, marginal R^2^, represent variance explained by fixed effects; R^2^_c_, conditional R^2^, represents variance explained by fixed and random effects.

## Data Availability

The original contributions presented in this study are included in the article. Further inquiries can be directed to the corresponding author.

## Supplementary Materials

The following supporting information can be downloaded at: https://www.mdpi.com/article/10.3390/jfmk11030362/s1, Table S1: Match and set participation of male volleyball players during the competitive season.
