# Supplementary material for: The Association Between Muscle Strength, Thigh Circumference, and Serving Efficiency in Elite Male Volleyball Players: A Prospective Observational Longitudinal Study
Source: J Funct Morphol Kinesiol. 2026 Sep 9;11(3):362. doi: 10.3390/jfmk11030362 (PMC13608733; doi:10.3390/jfmk11030362)
Supplement: Supplementary file 1 [file jfmk-11-00362-s001.zip › jfmk-4499605-supplementary.pdf]

Table S1. Match and set participation of male volleyball players during the competitive season*.

Player ID	Playing position	Matches played	Sets played
101	Setter	21	84
102	Setter	12	14
103	Middle blocker	18	66
104	Middle blocker	10	25
105	Middle blocker	20	80
106	Middle blocker	1	2
107	Opposite	11	14
108	Opposite	21	81
109	Spiker	21	82
110	Spiker	12	12
111	Spiker	6	9
112	Spiker	21	80
113	Libero	3	7
114	Libero	18	84
201	Spiker	5	12
202	Spiker	27	73
203	Middle blocker	18	47
204	Libero	26	83
205	Spiker	24	62
	Mean (SD)	15.5(7.9)	48.3(33.3)

* Matches played indicates matches in which the player participated; sets played represents the total number of sets played during the competitive season. ID= identification number; SD= standard deviation
